# The Guyana Program to Advance Cardiac Care: A Model for Equitable Cardiovascular Care Delivery

**DOI:** 10.5334/gh.1193

**Published:** 2023-04-27

**Authors:** Sheila L. Klassen, Karen Then, J. Wayne Warnica, Jennifer Burton, W. Orrin Stephen, Tanis Lane, Robert Dwhytie, Tracey DeBoice, Mahendra Carpen, Madan Rambaran, Filio Billia, Debra L. Isaac

**Affiliations:** 1 Program in Global Noncommunicable Disease and Social Change, Department of Global Health and Social Medicine, Harvard Medical School, Boston, MA, USA; 2 Center for Integration Science, Brigham and Women’s Hospital, Boston, MA, USA; 3 Libin Cardiovascular Institute of Alberta, Department of Cardiac Sciences, University of Calgary, Calgary AB, Canada; 4 Faculty of Nursing, University of Calgary, Calgary AB, Canada; 5 Diagnostic Cardiac Sonography Program, Mohawk College, Canada; 6 Department of Biomedical Engineering, Peter Lougheed Centre, Alberta Health Services, Canada; 7 Georgetown Public Hospital Corporation, Georgetown, Guyana; 8 Peter Munk Cardiac Center, University Health Network, Toronto, ON, Canada

**Keywords:** cardiovascular disease, global health, health systems, low-resource settings, Guyana

## Abstract

Guyana is one of the poorest countries in South America, with the highest rate of cardiovascular mortality on the continent. As is the case in many low- and middle-income countries, cardiovascular care is available through the private sector but is not accessible to much of the urban and rural poor. We present the 10-year experience of the Guyana Program to Advance Cardiac Care (GPACC), an academic partnership aiming to provide high-quality, equitable cardiovascular care in Georgetown’s only public hospital. We discuss the implementation of a cardiac care program using the World Health Organization Framework for Action, outlining vital components for care delivery in resource-limited settings. GPACC was able to demonstrate that targeted investment, education of clinicians, and cohesive healthcare delivery strategies can contribute to sustainable service delivery for Guyana’s largest burden of disease. This structured approach may provide lessons for implementation of similar programs in other resource-limited settings.

**Highlights:**

- In many LMICs, specialized cardiovascular care is available in the private, but not public, sector.
- The WHO Framework for Action can guide development of sustainable programs in low-resource settings.
- GPACC can serve as a successful and innovative model for delivery of sustainable cardiovascular care.

## Background

According to the Global Burden of Disease Study, cardiovascular disease prevalence is increasing worldwide, particularly in low- and middle-income countries (LMICs) [1]. While interventions such as integration of cardiac care units, utilization of evidence-based medication, and use of coronary angioplasty have caused a dramatic decline in cardiovascular death in high-income countries [2], availability of these interventions is limited elsewhere. Because these services are often only available through the private sector in LMICs, cardiovascular care is a major cause of catastrophic health expenditure or financial hardship caused by health payments [3]. The high cost of drug therapy alone results in millions of people worldwide being pushed into poverty [4].

**Figure 1:**
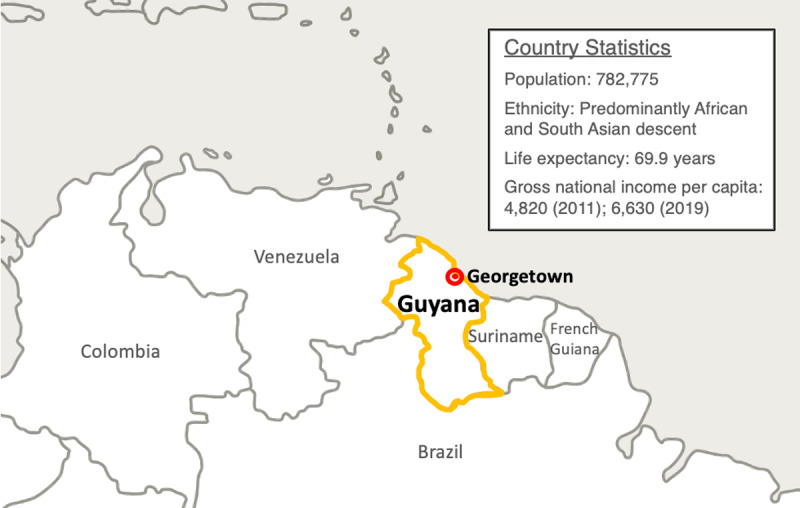
Map of the Northeastern portion of South America. Location of Georgetown, the capital city, is shown and country statistics are derived from The World Bank [6].

Guyana was, until recently, one of the poorest countries in South America [5], located on the North Atlantic coast (Figure 1). The prevalence of cardiovascular disease in Guyana is one of the highest in the Americas [7], with the highest rate of age-standardized disability-adjusted life years on the South American continent [1]. Disability-adjusted life years estimate the years of life lost due to premature mortality, years of life lost due to time lived in states of less than full health, or years of healthy life lost due to disability, adjusted for differences in the age distribution of the population. Guyana’s CVD rates exceed Canada’s by 2.7 times, with no evidence of decrease over the last 20 years [7]. Guyana has the highest CVD mortality in the Americas (291.9/100,000, compared to the average of 167.9/100,000) [8], and the standardized rates of cardiovascular mortality in Guyana are significantly higher than the global average [4]. In fact, ischemic heart disease is the leading cause of death in Guyana, while hypertensive heart disease ranks fourth [1].

This paper describes the 10-year experience of the Guyana Program to Advance Cardiac Care (GPACC), an academic partnership with the aim of providing high-quality pediatric and adult cardiovascular care in Georgetown’s only public hospital. The aim of GPACC is to address the high burden of cardiovascular disease in the large Guyanese population who cannot access cardiac care and provide compassionate and equitable care for the urban and rural poor. We discuss the implementation of GPACC using the World Health Organization Framework for Action, also used by other academic global health partnerships [9], which outlines vital components of a health system and provides a clear way to define health systems strengthening. It served as a way for us to describe the various aspects of GPACC development and how they contribute to overall health systems strengthening in Guyana.

## The Guyanese Health System and Cardiovascular Care Prior to GPACC

In the current Guyanese health system, medical care is concentrated in the capital city of Georgetown. Access to services by Guyanese who live inland, predominantly Indigenous populations, is made difficult due to poor road infrastructure and dense forestation. Healthcare provider capacity and availability of equipment are limited in the public sector, forcing patients to pay out of pocket for private tests and services if they have the means to do so. In the public system, availability of anything beyond basic diagnostic testing (such as common laboratory investigations) has been sporadic at best, though slowly improving over time through partnership with nongovernmental organizations. Patient documentation is paper-based, and no routine quality assurance measures exist. The public healthcare insurance system provides very limited coverage and does not include medication coverage. The amount of cardiovascular disease training integrated into general nursing and medical school programs is minimal, thus heart disease recognition and management in general medical units of the public hospital is poor.

Georgetown Public Hospital Corporation (GPHC) is a high-volume publicly funded teaching hospital and national referral center. As the largest government-run hospital in the country, an estimated 75,000 patients are cared for in the emergency department yearly, but only basic cardiac care existed prior to GPACC. Cardiac diagnostic equipment consisted of only intermittently functioning electrocardiogram (ECG) machines in the emergency department. There were no echocardiography machines and no monitored beds or defibrillators outside of the medical intensive care unit. Heart failure was primarily treated with furosemide monotherapy. Streptokinase was administered intravenously for myocardial infarction in the emergency department on occasion, but interpretation of ECGs amongst emergency physicians was variable, and no cardiac stress testing or coronary angiography was available. Access to a cardiac specialist was very limited within the public healthcare system. If patients or families had the means, cardiologist consultation was sought at a private facility in Georgetown or overseas. At times, application for funding for cardiac consultation from governmental or charitable sources was successful but was available only to a small proportion of those in need.

## The Formation of an Academic Partnership between Libin and GPHC

In November 2011, a Guyanese cardiac surgeon working in Calgary, Alberta, Canada, requested donations from the Libin Cardiovascular Institute of Alberta (LCIA) for echocardiographic equipment in order to improve cardiac care in his home country. LCIA is a joint entity of the local health system and university (Alberta Health Services and the University of Calgary) and coordinates all research, education, and cardiovascular care delivery services in the region. Prompted by this request, an initial visit to Georgetown by members of the LCIA team that would later become GPACC revealed the lack of, and critical need for, publicly accessible and high-quality cardiac care. However, the absence of a health workforce familiar with cardiac care and use of echocardiography equipment was also apparent. An opportunity presented to form an academic partnership with the public sector to implement a comprehensive model of cardiac care. Conversations were initiated between faculty at LCIA and the Minister of Health, Chief Medical Officer at the Ministry of Health, Chief Executive Officer (CEO) of GPHC, and the Director of the Institute of Health Sciences Education (IHSE) at GPHC.

## First Steps: The Formation of the Echocardiography Education Program

In partnership with national and hospital leadership, LCIA launched an echocardiography education program based on American Society of Echocardiography (ASE) standards, aiming to teach physicians to perform and interpret comprehensive transthoracic echocardiograms. Volunteer physicians and cardiac sonographers from LCIA and Mohawk College in Ontario, Canada, developed a training curriculum, arranged for donation and shipping of full-service used echocardiography machines in good working order (Philips HP5500 and HP7500) and performed hands-on training with Guyanese physicians.

This echocardiography education program was initially structured as an annual three-month program of didactic PowerPoint lectures, written tests, and hands-on scanning delivered primarily in person (available upon request). Curriculum material was written and designed by experienced echocardiographers from LCIA and adapted for context. Cloud-based image review software allowed for remote supervision after completion of the initial training program. Echocardiographers at LCIA reviewed most studies performed by trainees and provided regular and ongoing feedback to trainees for skills development. Additional on-site refresher and advanced training was then provided four months after completion of the initial program. This primarily consisted of additional materials on congenital heart disease as well as addressing knowledge and skills gaps identified during interim virtual mentorship.

An early pivotal moment was the recognition that correlation between echocardiograms and the clinical status of the patient was necessary, and that there was no referral pathway for clinical management for echocardiographic findings at GPHC. This realization occurred over the several months following the initial cohort of trainees completing the echocardiography education program, as case numbers increased. This led to plans to develop a comprehensive cardiac care program now known as GPACC.

## Development of GPACC’s Comprehensive Cardiac Care Program

The majority of patients referred for echocardiography presented with clinical heart failure and had evidence of structural heart disease on imaging. Thus, a natural progression in program development was implementation of a heart function clinic (HFC) adjacent to the echocardiography laboratory. Selected nurses nominated by GPHC Nursing Administration and the Director of Medical and Professional Services were trained to provide volume status assessments and heart failure management according to Canadian Cardiovascular Society guidelines [10]. Education and mentorship of these nurses was provided by senior HFC nurse clinicians from LCIA both in person and via regular videoconferences and case review.

Referrals for echocardiography revealed high numbers of children with cyanotic and complex congenital heart disease who were undiagnosed and unrepaired. This recognition led to a partnership with an international pediatric cardiac services foundation. However, corrective surgery for congenital defects meant that a pediatric intensive care unit was necessary for post-operative care. Local physicians and nurses were trained to operate this unit with locally sourced and donated equipment and medications. Local pediatricians were trained in pediatric echocardiography, clinical evaluation, and management of congenital heart defects, as well as pre- and post-operative management of these children.

There was a growing number of patients from the echocardiography laboratory identified with acute in addition to chronic heart disease. With the lack of existing cardiology services to address this problem, a designated inpatient and outpatient cardiology service was developed. Frequent onsite visits from GPACC cardiologists and nursing educators with weekly remote educational sessions allowed for timely remote and in-person case reviews. These factors further allowed for the development of a cardiac intensive care unit (CICU) at GPHC in 2016. Since no cardiac nursing positions existed in Guyana, concurrent establishment of a cardiac nursing training program was required.

To further support the provision of cardiac care at GPHC, GPACC sponsored the development of an ECG and stress testing lab in 2016. Provision of additional cardiac diagnostic services (exercise and pharmacologic stress testing, exercise echocardiography, inpatient and outpatient ECGs) were progressively introduced.

**Figure 2:**
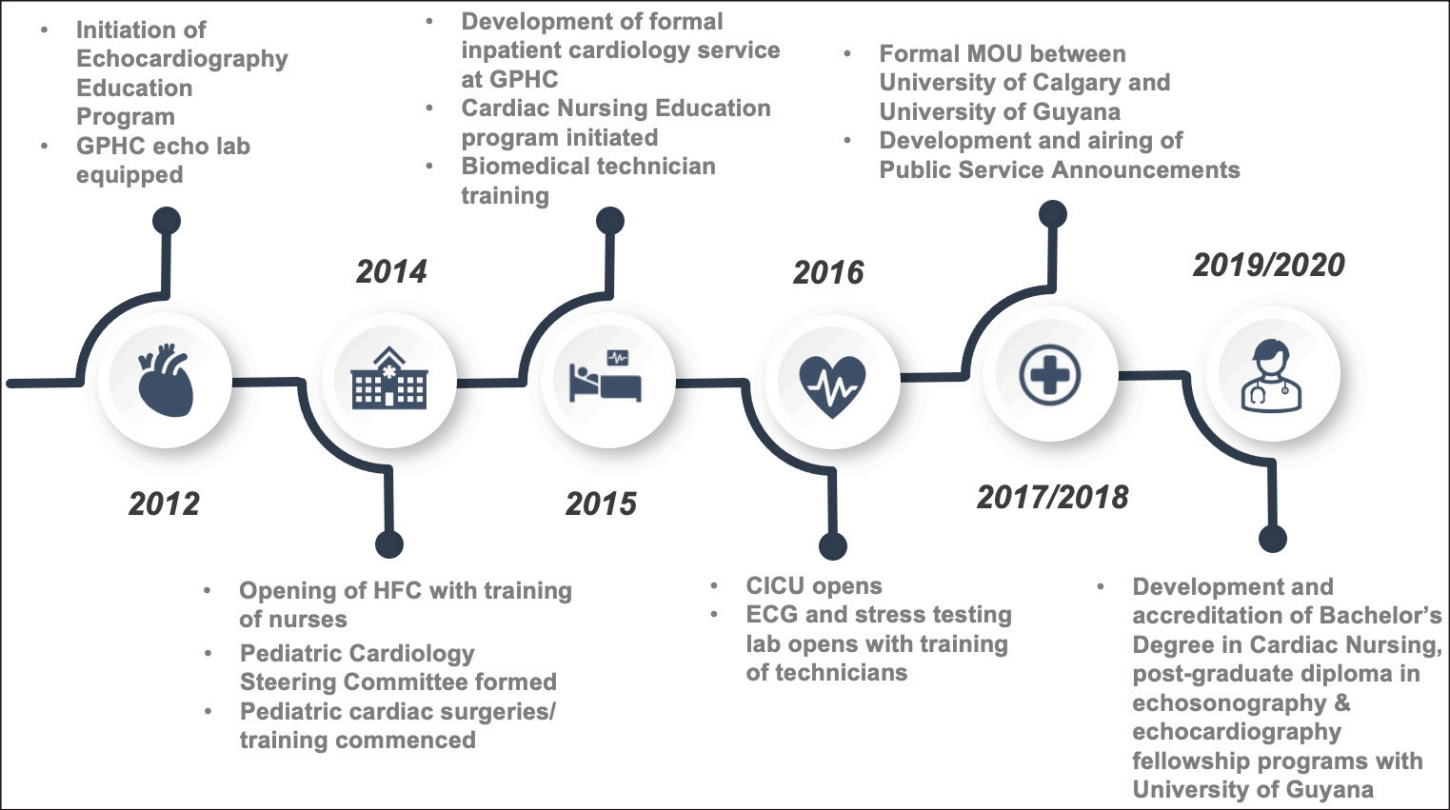
Timeline with key milestones in GPACC’s development. CICU, cardiac intensive care unit; ECG, electrocardiogram; GPHC, Guyana Public Hospital Corporation; HFC, heart function clinic; MOU, memorandum of understanding.

Each milestone in GPACC’s development (Figure 2) was prompted by a recognized gap in service delivery and by personal encounters between GPACC or GPHC staff and patients in need of services unavailable in Guyana’s public healthcare system. Each milestone was supported by the Guyanese government and hospital leadership with further commitment in funds and human resources.

Once inpatient and outpatient programs were initiated, it became clear that health literacy and awareness of cardiac disease and risk factors was very poor in the general Guyanese population. Prior studies have also found that patient delays in seeking medical attention attributed to lack of recognition of symptoms were a major cause of poor outcomes in myocardial infarction in LMICs [11]. This led to the development of public service videos, sponsored by GPACC and a Canadian media production company, and supported by the Ministry of Public Health of Guyana. These videos are run on local television stations to inform the public about symptoms of heart disease, appropriate response to these symptoms, and the availability of cardiac care at GPHC independent of personal financial resources.

## System building blocks and current GPACC operations

**Figure 3:**
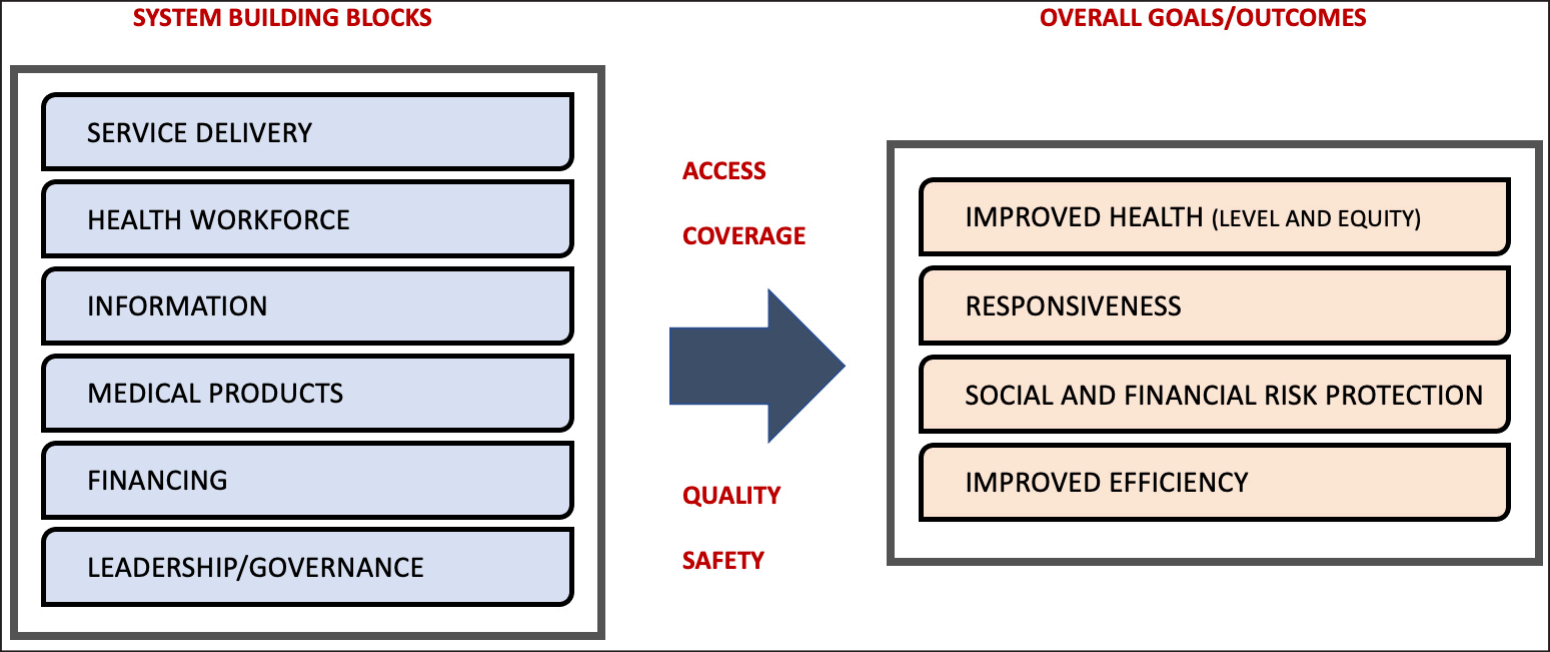
The World Health Organization Health Systems Framework. System building blocks are integrated to achieve desired goals and outcomes. Adapted from the World Health Organization [9].

There are six essential ‘building blocks’ outlined by the World Health Organization which describe basic functions required by health systems to achieve their goals of improved health, responsiveness, social and financial risk protection, and improved efficiency (Figure 3). These building blocks are interconnected and must function together in order to be effective. The Framework is based on the premise that comprehensive health systems strengthening in resource-limited settings is the only way to secure better health outcomes [12]. These were adapted to GPACC as they also functioned well to describe and guide development of a comprehensive cardiac care program.

### Service delivery

The Echocardiography Education Program and echocardiography laboratory has enrolled 20 physicians and seven cardiac sonographers over the last 10 years, though some have not been able to complete the program due to high standards for certification, set to ensure quality of care. Retention of echocardiography staff has been challenging due to lack of skills retention, emigration out of Guyana, and movement into the private sector. The remainder of trained physicians and sonographers continue to scan at GPHC, two with a pediatric focus. Echocardiograms continue to be reviewed remotely to provide expert telemedicine consultative services, and over 50 international sonographers and nine cardiologists have supported this education program. Over 10,000 transthoracic echocardiograms have been performed to date through this full-service echocardiography laboratory, free of charge to patients.

The original program has been formalized and split in two: a one-year postgraduate diploma in echosonography for students with background training in medical imaging, nursing, and allied health, and a one-year fellowship in echocardiography for physicians trained in cardiology, internal medicine, or pediatrics, certified through the University of Guyana and IHSE. The physician program includes training in advanced modalities such as stress echocardiography, transesophageal echocardiography, and echocardiography-guided procedures such as pericardiocentesis.

An estimated 250 patients are currently seen per week in outpatient cardiology clinics and approximately 700 patients are registered in the pediatric database. This includes children and young adults awaiting surgical evaluation or intervention for congenital lesions. The database serves as a tool to track ongoing follow-up of these patients.

The CICU consists of 14 monitored beds and is usually at over 90% capacity. There is an inpatient cardiology consultation service on the medical wards for patients not requiring CICU level support. Demographics of admitted GPACC patients between 2015-2021 are detailed in Table 1. The majority of primary diagnoses consisted of acute coronary syndrome and clinical heart failure, but specific admitting diagnoses cannot be easily tabulated due to the design of the electronic record. Since 2016, GPHC has been able to provide cardiac catheterization and coronary intervention to all high-risk cardiac patients according to medical need (1,031 and 635 procedures, respectively). There are now over 2,000 patients who are managed and follow on an ongoing basis with the HFC, and a new cardio-obstetrics clinic has been launched due to population need.

**Table 1:** Description of admitted GPACC patients from 2015–2021.


	(n = 2264)

Mean age in 2021, years (SD)	62.4 (14.4)

Female (%)	1078 (48)

Number of hospital admissions	2906

Median duration of hospital admission, days	4

Mean ejection fraction on echocardiogram, % (n = 1954)	39

Deaths recorded in hospital	12


Demographics of admitted GPACC patients were downloaded from the online GPACC database (established in 2015); GPACC, Guyana Program to Advance Cardiac Care.

In the ECG and cardiac stress testing lab, there are currently approximately 60 ECGs performed per day and 574 treadmill stress tests performed to date, all free of charge to patients.

### Medical products

As is common in many LMICs, the hospital pharmacy at GPHC provides medication for patients free of cost but is often and unpredictably stocked out of essential medicines due to challenges with procurement. As part of clinical care during the first several years of the program, clinicians asked patient family members to access pharmacies in the city and pay out of pocket for medications essential to outpatient and inpatient cardiac care. Guyana does not have a national program for medication coverage leaving patients of little financial means and rural patients vulnerable to poor outcomes resulting from inconsistent medication use.

To combat this problem, a small pharmacy was initially established within the CICU. Clinical cardiac pharmacists from LCIA worked directly with local GPHC pharmacists to provide clinical education and mentorship regarding the role that pharmacists play in clinical management and advocacy. A more consistent supply of medications is now available, including medications required in the CICU, such as intravenous milrinone, nitroglycerin, and labetalol, oral bisoprolol and enalapril, and sublingual nitroglycerin. Medications are dispensed according to medical needs of patients without relying on factors for allocation. However, outpatient mechanisms for medication access and coverage continue to pose challenges leading to decreased medication adherence and dosing consistency across GPACC outpatient programs.

To date, donations of major medical equipment required for GPACC operation have included cardiac diagnostic equipment (such as CICU cardiac monitoring system and ECG machines) and medical equipment required for critical care (such as ventilators and equipment for cardiopulmonary resuscitation/‘crash carts’). Other non-medical equipment donations are also essential to operation of cardiac units and the echocardiography laboratory, including computers for medical documentation, servers for image archiving, power supply units to provide uninterrupted power given frequent electrical outages, and specialized high-quality video monitors for echocardiography interpretation. The AccessPoint image review and archiving system was provided by Freeland Systems LLC (Indiana, USA). This remote echocardiography reading system is essential for virtual case review, which has strengthened knowledge and confidence of Guyanese providers over the course of evolution of the GPACC program.

Given the amount of essential equipment necessary for the ongoing provision of cardiac care, it became clear that equipment maintenance and repair was critical to the ongoing operations of cardiology services at GPHC. Servicing of cardiac equipment, such as echocardiography machines, requires high-level training and experience, but none of the necessary biomedical support was available locally. This led to the involvement of a senior biomedical professional from Alberta who has undertaken frequent onsite training visits and provides ongoing remote support. This has resulted in a significant improvement in management and maintenance of cardiac equipment at GPHC.

### Health workforce

The philosophy of GPACC has always been to improve access and standard of cardiac care through education, and this philosophy continues to be prioritized to ensure long-term sustainability of the program. Developing the health workforce for GPACC meant training all clinical providers in the basics of cardiology given the lack of dedicated cardiac training programs in the country. Robust channels of telecommunication were developed for ongoing mentorship in clinical care, continuous skills development, and ongoing relationships when the LCIA team and other international faculty were not in-country.

Training occurs at GPHC embedded within clinical care in the form of bedside rounds and other patient care scenarios. Additional forms of training include weekly remote ECG teaching sessions for medical officers, in-person skills training for all clinical providers, and annual re-certification for CICU nurses in arrhythmia interpretation, defibrillation, intravenous cardiac drug administration, physical examination, and cardiac arrest procedures. Basic and advanced cardiac life support and cardiopulmonary resuscitation courses are provided by GPHC. Recertification is performed by LCIA faculty with the plan to transition recertification to a Guyanese cardiac nurse educator trained by GPACC. Physicians and echosonographers in the GPHC echo lab must meet ASE standards. Annual continuing medical education is mandated and provided through attendance (in person or virtually) at ASE educational programs.

The cardiology service is currently staffed by internal medicine-trained government medical officers (equivalent to the level of a junior residents), registrars (equivalent to a senior resident), and medical learners. Early program development from 2012 onward was supported by the return of a Guyanese physician trained in adult cardiology with subspecialties in coronary intervention and electrophysiology in Canada and the United States. This cardiologist worked predominantly in the private sector but eventually became the co-director of GPACC. He took on additional administrative and leadership roles within the program and is now the Head of Cardiology at GPHC. A Guyanese cardiologist who completed a full adult cardiology and interventional cardiology fellowship at LCIA return to work at GPHC in 2021 and another adult-trained Guyanese cardiologist returned to Guyana in 2022.

The cardiology nursing program initiated in 2016 now provides registered nurses with a University of Guyana bachelor’s degree program in cardiovascular nursing. There are 28 nurses who have successfully completed the one-year cardiac certification nursing program though many have left GPACC. Prior to GPACC, nurses did not receive any cardiac-specific training during their general nursing programs. GPHC has also supported the initiation of a Guyanese cardiac nursing educator for GPACC.

There was recognition in early stages that local coordination for the echo program and subsequent expanded GPACC activities was critical. In 2014, a local administrative coordinator, recommended by the CEO, was hired using start-up GPACC funds. This coordinator manages activities of the echo lab, coordinates educational programs, and oversees the database and bookings for pediatric cardiac patients. Hospital leadership has recognized the importance of administrative support, and GPHC has since assumed the salary of this coordinator as well as other clerical assistants in the echo lab and HFC.

**Figure 4:**
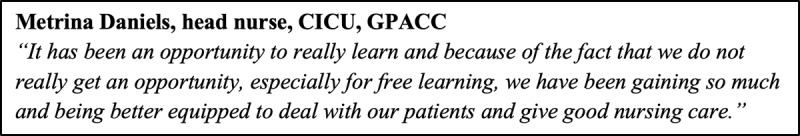
Quotation by Metrina Daniels. Quotation taken from the Switching Rhythm documentary film.

Hiring of GPACC staff is a shared effort between GPACC faculty, human resources at GPHC, and the CEO of GPHC. Staff absenteeism has been a challenge due to the lack of consequences for such absenteeism. There are ongoing challenges in retaining nurses trained to practice in the CICU and HFC. Poor remuneration at GPHC cause staff to pursue better-paying positions elsewhere once fully trained. There is a high rate of emigration out of Guyana and movement into the private sector. In addition, nurses remain employees of the hospital and due to lack of recognition of the significance of specialty nursing training, cardiac-trained nurses may be intermittently deployed to other units such as obstetrical wards. Staff retention significantly impacts program integrity, and small stipends for successful nursing training helps cardiac-trained nurses to remain in the CICU where their skills were best utilized. Healthcare providers trained within GPACC have expressed appreciation (Figure 4) and nurses who do successfully complete the cardiac certification program are very involved in training others.

The GPACC faculty has made it a priority to model professionalism, fostering teamwork with physicians and nurses working in a partnership of mutual respect, and to ensure that local staff are recognized for their roles in the provision of a high standard of cardiac care in GPHC. Among GPACC staff, development of expertise and recognition of their contribution has helped to instill a sense of pride in their work and has helped to improve provider retention. There is potential for additional formalized structures to promote specialized education including opportunities for career advancement.

### Health Information

Paper charting for medical record keeping remains standard across GPHC and in many institutions in Guyana. However, paper charts are difficult to access in the health records department and are easily damaged. Prior to GPACC, cardiac patients who frequented the hospital often did not carry medical records or medication lists with them. The course of prior hospitalizations of each patient was dependent on the memory of the medical officers who were present at the time.

An online password-protected database was designed in 2015 specifically for GPACC adult outpatients and inpatients, providing an easily accessible method of tracking medication regimens, prior tests results including echocardiography results, and prior hospitalizations. Nurses and medical officers use encounter forms embedded within the database to record clinical information. While not a formalized electronic medical record, it functions as a secure repository for patient data accessible at point-of-care, though the data points gathered are limited to facilitate ease of use at the bedside.

Data from inpatient admissions was used to demonstrate success of GPACC in improving adherence to evidence-based heart failure medications and decreased mortality, even in the early years of program implementation [13]. The database remains a rich resource for monitoring and evaluation of program outcomes. The major challenge to the online database remains inconsistent hospital internet availability and power outages. However, advantages of the online system include its availability on smartphones over cellular data as well as accessibility remotely by the LCIA team for mentoring, quality assessment, and research. Use of the database has led to numerous conference abstracts and publications reporting on the GPACC experience.

Successful programs are often not simply contingent on financing, supply of medical products, and a large health workforce but by the way these components are integrated. Health information systems and ongoing quality assessment are critical in evaluating effectiveness of care delivery and improving system efficiency.

### Health Financing

At GPHC, public hospital and care spaces, salaries, and utilities are provided by the hospital with government administration. Prior to GPACC, inpatients would travel from GPHC to private facilities to access imaging such as computed tomography, or family members would bring blood samples drawn at GPHC to a private lab for testing if these services were not available in the public hospital. A limited public health insurance system exists to directly reimburse basic medical costs, but this does not affect service provision in the public healthcare system and is not adequate for structural reform. Currently, invasive cardiac procedures by GPHC cardiologists committed to working within the public system is covered under a Ministry of Health budget subject to volume-negotiated rates, where previously this was only available in the private sector.

GPHC’s limited budget historically had not been able to fund specialized cardiac diagnostics or interventions. Much of GPACC’s start-up costs were funded through program development grants from LCIA, research grants through LCIA and the University of Calgary, a grant through the Libin Foundation for videoconferencing equipment for telemedicine and mentorship, a vocational training grant from Rotary International, donations from Canadian hospitals, and other smaller donations. Granting organizations, whose stipulations for funding have always included criteria for sustainability through education, has further solidified GPACC as a program founded on training to improve health systems. These funds covered medical equipment purchase and donations, training equipment and materials, and travel costs for international volunteers and faculty. Investment since program conception totals over 1.1 million Canadian dollars.

Currently, all local staff are salaried by GPHC, who is continuing to hire for the GPACC program. GPHC also independently financed the physical conversion of a prior hospital ward space into an operational CICU. In addition, ongoing maintenance and equipment replacement costs will be assumed by GPHC as well as any necessary physical space upgrades.

Resources in the public sector of most LMICs is scarce and creative modes of financing are required. GPACC continues to accept all patients regardless of their financial circumstances and this model has remained acceptable to the Ministry of Health of Guyana. Beyond initial start-up capital, which can be provided as one-time charitable donations and grants, ongoing investment, income generation, and payment for service provision remains a major challenge for many programs like GPACC in low-resource settings. Recognition of, and commitment to, local health sector investment is needed to ensure continuity of programs as GPACC international faculty assume a progressively lesser role in future program activities.

### Leadership and governance

From the beginning, the GPACC team realized the importance of a bilateral commitment between external faculty and local leadership. Initial implementation meetings included all national, academic, and institutional stakeholders (Table 2). Engagement from the Head of Medicine and Head of Pediatrics was obtained shortly after program implementation, which impacted decisions regarding subsequent service delivery. At every progressive juncture in GPACC’s development, consultation and joint decision making with these major stakeholders was sought, complexities in local service delivery were discussed, and bilateral commitment was obtained. Each milestone depicted in Figure 2 was preceded by meetings with these stakeholders to gain approval for expansion of the program and to plan for an increase in services. There are now local clinical leaders designated for the echocardiography laboratory, CICU, and pediatric cardiology who are assuming an increasing role in governance of their respective programs as has been mentioned in previous sections.

**Table 2:** Guyanese leadership and stakeholders engaged in GPACC implementation.


LEADERSHIP AT GPHC*	LEADERSHIP OUTSIDE GPHC*

CEO Director of Medical and Professional Services Head of Medicine Head of Pediatrics Human Resources Nursing Administration	CEO at the Ministry of Health Dean of Health Sciences at the University of Guyana & associated university leadership Director of the Institute of Health Sciences Education Minister of Health


* Listed alphabetically. GPHC, Georgetown Public Hospital Corporation; CEO, chief executive officer.

As it became clear that education needed to be formalized to strengthen programs and improve sustainability, in addition to the ongoing relationship with the Director of IHSE, GPACC collaborated with the Dean of Health Sciences at the University of Guyana and associated university leadership. There is a Memorandum of Understanding between the University of Calgary and the University of Guyana for educational programs, and GPACC is a program incorporated within the Indigenous, Local, and Global Health Office at the Cumming School of Medicine, University of Calgary.

All GPACC programs are regularly reviewed by IHSE, and international faculty continue to provide oversight and review the quality of clinical care and echocardiography. On-site visits for review of CICU patient care now occurs at least three times per year by senior cardiologists from Canada. Locally trained Guyanese educators in the CICU, echo lab, and HFC lead active quality assurance practices.

## Lessons Learned and Future Directions in GPACC’s Development

Development of cardiovascular care to a high standard is ongoing work with evolving collaborations and continuous training efforts. The building blocks of the World Health Organization Framework are interdependent, and it was difficult to separate descriptions of program elements into sections for the writing of this review. This realization not only catalyzed change within the program but influenced the strategy in GPACC’s development. There are very few published reports in the literature describing implementation of comprehensive cardiovascular care programs, particularly programs providing acute and critical cardiovascular care in resource-limited settings. Moreover, lack of critical care infrastructure has been identified as a barrier for acute myocardial infarction care in LMICs [11].

Relationship building was critical among mentors and mentees, and substantial face-to-face time in addition to ongoing, sustained remote communication contributed to the building of trust and setting of expectations among all team members. Healthcare worker education and feedback has been identified by multiple prior studies as an effective implementation strategy for health systems strengthening in low-income countries [14]. Workforce development was critical in the sustainability of this project but retention in Guyana’s public system remains a challenge. A priority for GPACC is to ensure consistent onsite and remote faculty support for Guyanese providers with the hopes that expert providers will remain in the public sector. Recent trainees of the GPACC program are now becoming educators and leaders within the program, bolstering local, context-driven expertise. The eventual goal is a transition in leadership from the LCIA founders of GPACC to local leaders and program staff who will continue to enrich its services and expand its reach.

Strategies that have contributed to the strengthening of GPACC include institutional recognition of increased staff expertise, support for training and maintenance of competencies, and robust quality improvement initiatives. Local champions, particularly clinician leaders, are essential to driving program-building efforts and providing context to implementation. The establishment of GPACC within GPHC has underscored the importance of a high-quality, team-based approach to care.

## Conclusion

GPACC remains a work in progress but has provided equitable, publicly accessible, and high-quality cardiovascular care to thousands of Guyanese in a country with exceedingly high rates of cardiovascular morbidity and mortality. Importantly, the success of GPACC has demonstrated that targeted investment, education of healthcare providers, and cohesive healthcare delivery strategies can create durable health system structures for Guyana’s largest burden of disease.

**Figure 5:**
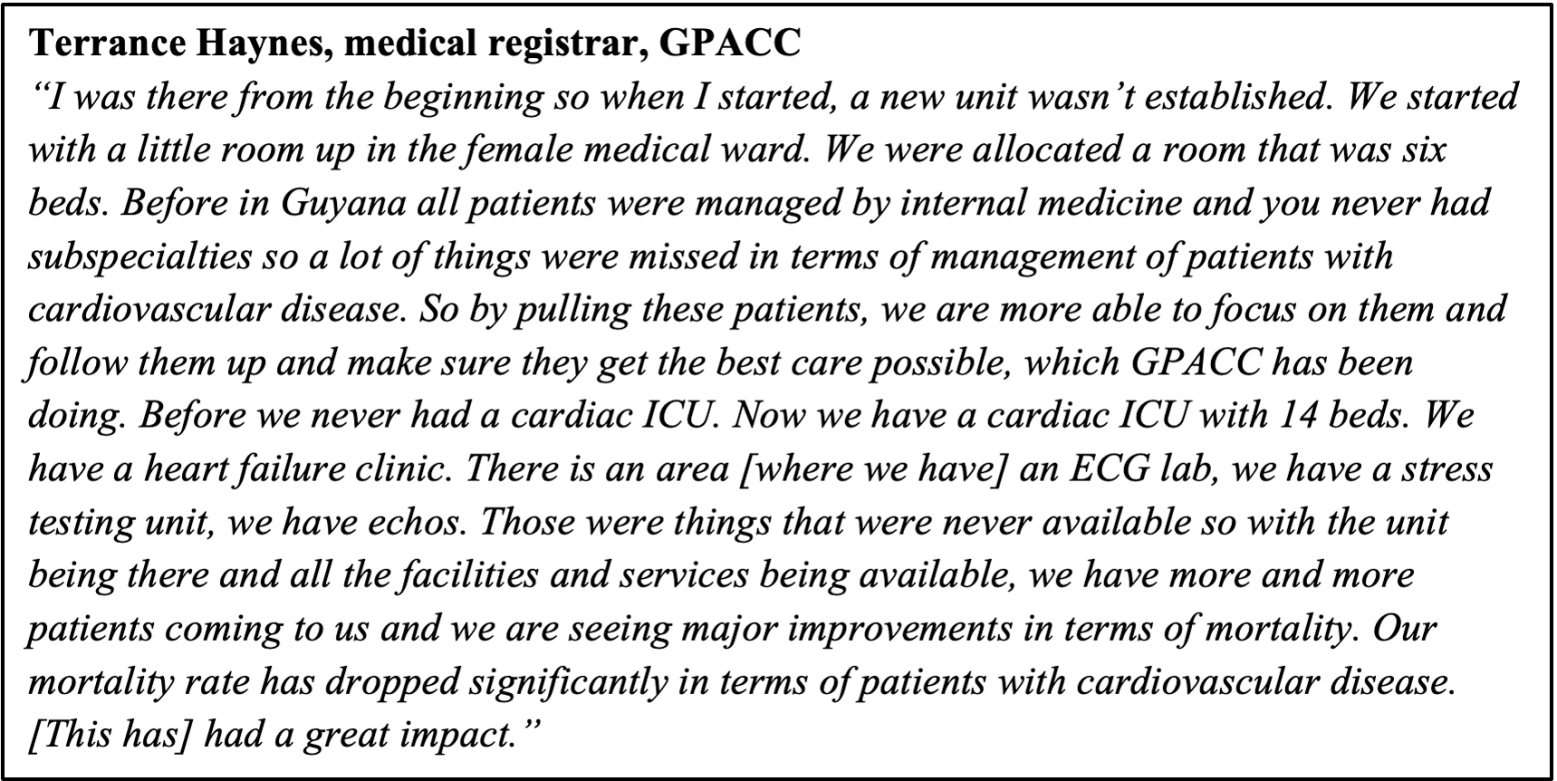
Quotation by Terrance Haynes. Quotation taken from the Switching Rhythm documentary film.

This ambitious project has grown from the tireless efforts of almost 100 international clinician and non-clinician staff and volunteers working toward the common goal of better cardiovascular care for the Guyanese people (Figure 5).

**Figure 6:**
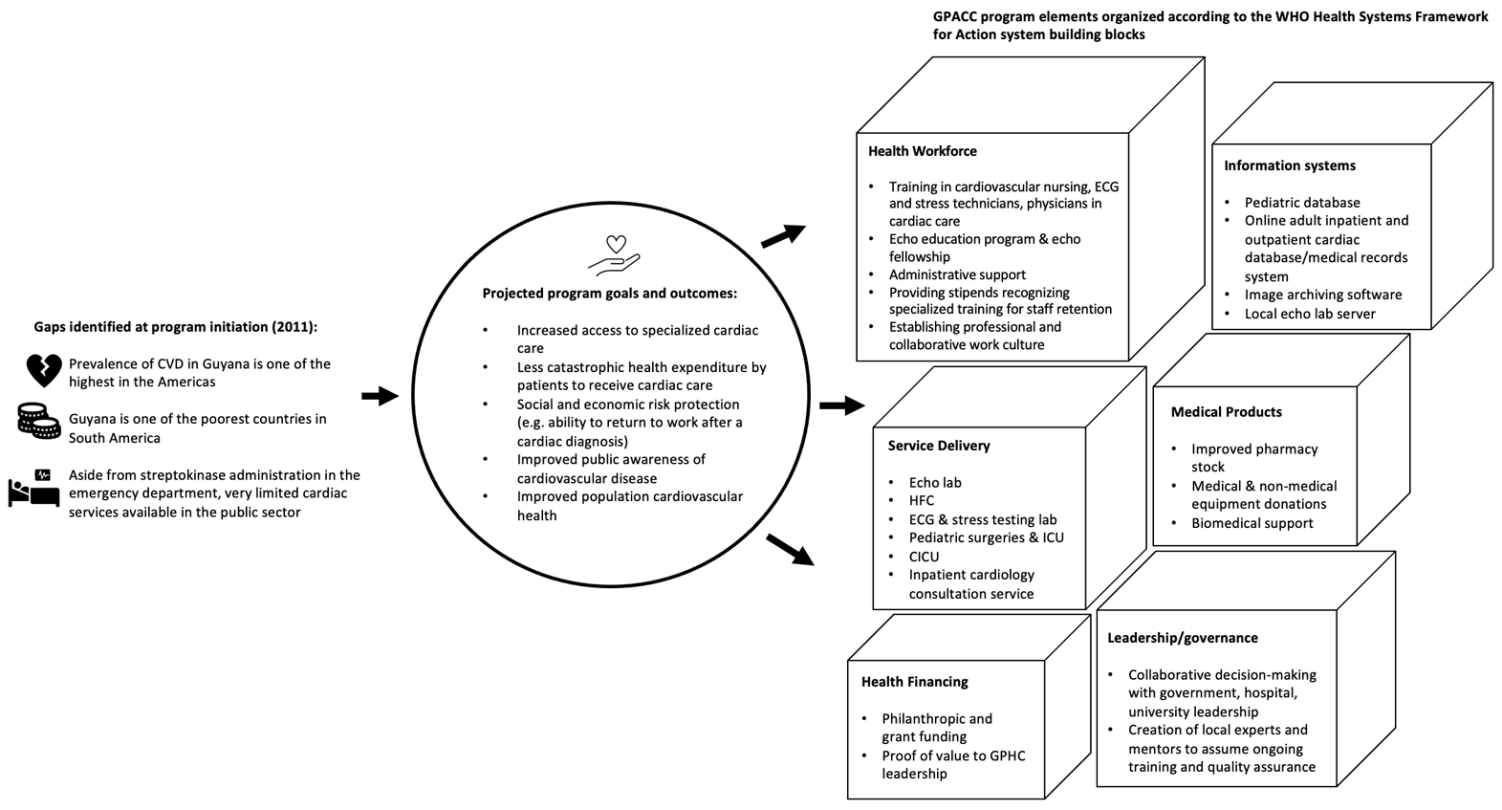
Outputs of GPACC using the WHO Framework for Action in response to identified program goals.

Using the World Health Organization Health Systems Framework for Action, we describe the building blocks of a dedicated cardiovascular care program established in Georgetown, Guyana, and how an academic and public sector partnership can lead to a successful cardiovascular care program in a lower middle-income country (Figure 6). GPACC’s firm belief is that high out-of-pocket expenditures leading to inequitable access to essential cardiac care is unjust and leads to poorer overall population health. The creation of GPACC not only addresses this service gap directly but creates a model for other Guyanese disease programs and contributes to overall health systems strengthening in the country. Methods for building capacity in each country context will differ but we believe that this experience and lessons learned can be valuable in similar programs based in resource-limited settings.

## Data Accessibility Statement

Switching Rhythm, the documentary film on GPACC produced by Mr. Stephen Warnica and the staff at Project 7 Media Group, is available at https://vimeo.com/250577099.

Training materials from the Echocardiography Education Program can be requested from the corresponding author.
